# Orchestrated Domain Movement in Catalysis by Cytochrome P450 Reductase

**DOI:** 10.1038/s41598-017-09840-8

**Published:** 2017-08-29

**Authors:** Samuel L. Freeman, Anne Martel, Emma L. Raven, Gordon C. K. Roberts

**Affiliations:** 1 Department of Chemistry and Leicester Institute of Structural and Chemical Biology, University of Leicester, Henry Wellcome Building, Lancaster Road, Leicester, LE1 7RH UK; 2 Institut Laue-Langevin, 71 avenue des Martyrs, 38000 Grenoble, France; 3 Department of Molecular & Cell Biology and Leicester Institute of Structural and Chemical Biology, University of Leicester, Henry Wellcome Building, Lancaster Road, Leicester, LE1 7RH UK

## Abstract

NADPH-cytochrome P450 reductase is a multi-domain redox enzyme which is a key component of the P450 mono-oxygenase drug-metabolizing system. We report studies of the conformational equilibrium of this enzyme using small-angle neutron scattering, under conditions where we are able to control the redox state of the enzyme precisely. Different redox states have a profound effect on domain orientation in the enzyme and we analyse the data in terms of a two-state equilibrium between compact and extended conformations. The effects of ionic strength show that the presence of a greater proportion of the extended form leads to an enhanced ability to transfer electrons to cytochrome *c*. Domain motion is intrinsically linked to the functionality of the enzyme, and we can define the position of the conformational equilibrium for individual steps in the catalytic cycle.

## Introduction

The concept of an energy landscape for a folded protein requires that proteins exist as an equilibrium population of conformational states. Interconversions between these states are of fundamental importance to biological function^1–4^, but remain incompletely understood. Internal motions in proteins range from bond vibrations through local loop movements to large-scale domain motion and occur across an extremely wide range of time scales (femtoseconds to seconds). The choreography of local loop motions in the catalytic cycle has been studied in some enzymes^5–8^, but in other instances much larger-scale movements of whole domains are important^9–11^. This is particularly true in electron transfer pathways; electron transfer (ET) is generally carried out by proteins associated in large dynamic complexes, and in such systems domain motion can be required to provide access for the protein partner(s) to the redox centre(s)^12–15^.

**Figure 1:**
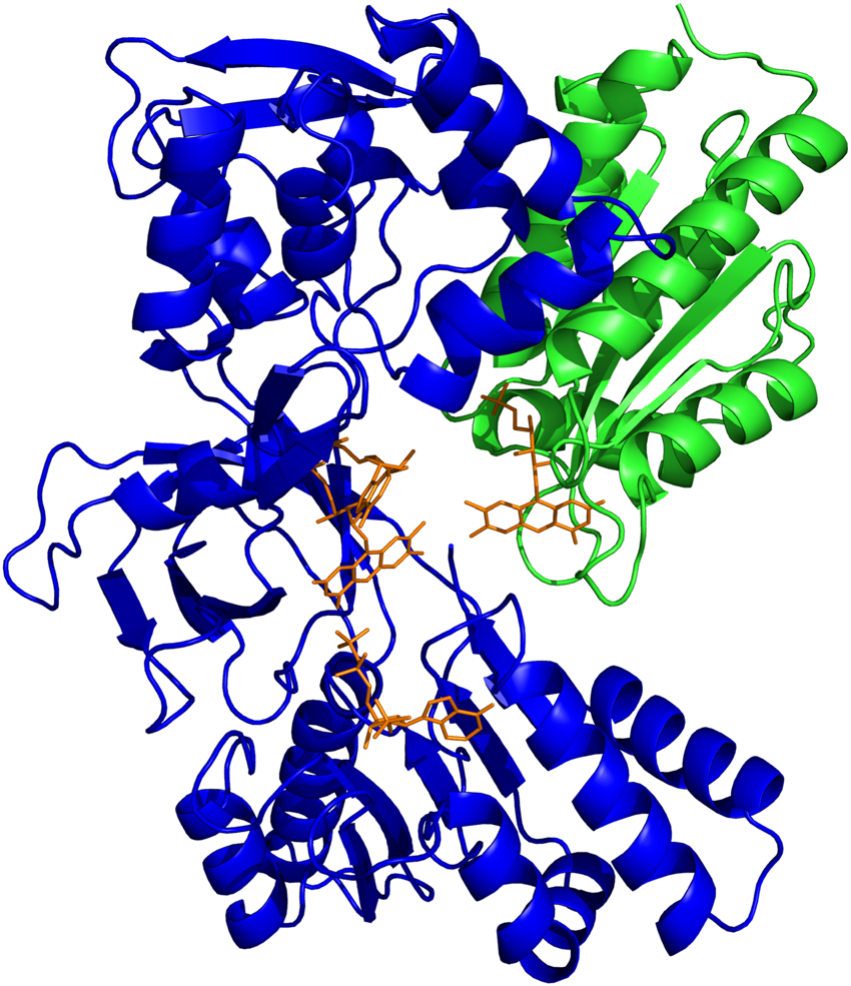
Crystal structure of human CPR in the oxidised state^19^; the FMN-binding domain is in green, the FAD-binding and linker domains in blue and the cofactors are shown as orange sticks.

An important family of ET proteins which depends on domain movement in this way is that of the diflavin reductases^16^, which includes cytochrome P450 reductase (CPR; Fig. 1)^17–19^, mammalian nitric oxide synthase (NOS)^20, 21^, the cancer-related novel reductase 1^22^, and methionine synthase reductase^23, 24^, as well as the bacterial proteins sulfite reductase^25^ and CYP BM3^26^. These enzymes (or their reductase components) have three domains: an FMN-binding domain, related to flavodoxins, an FAD- and NADPH-binding domain, related to ferredoxin/flavodoxin reductases, and a ‘linker’ domain, which may serve to position the other two domains. The FMN domain is connected to the linker and FAD domains through a highly flexible ‘hinge’. In all the members of this family the ET pathway involves the sequence NADPH → FAD → FMN → acceptor, and there is good evidence from mutagenesis and kinetic experiments for motion of the FMN-binding domain to allow it to accept electrons from the FAD and deliver them to the acceptor^18, 20, 21, 27–29^.

CPR is located on the endoplasmic reticulum where it is a key component of the P450 mono-oxygenase system which plays a central role in drug metabolism^18^, and in the biosynthesis of secondary metabolites in plants^30^. In man, polymorphisms or mutations in CPR can lead to changes in drug metabolism^31, 32^, and to disordered steroidogenesis and skeletal malformations^33, 34^. The conformation of truncated soluble CPR seen by X-ray crystallography^17, 19^ (Fig. 1) is well suited for rapid electron transfer from FAD to FMN, as the two isoalloxazine rings are ~4 Å apart. However, ET between the FAD and FMN co-factors is in fact relatively slow (10–60 s^−1^)^27, 28, 35, 36^, suggesting that the interflavin ET is ‘gated’. Furthermore, in the CPR conformation seen in the crystal it is difficult to see how cytochrome P450 (or cytochrome *c*, widely used as a surrogate redox partner for studies in solution) could approach close enough to the FMN for ET to occur^16, 18^.

There is thus evidence for the existence of domain motion in CPR and for its importance in catalysis^16, 18, 28, 37, 38^, but it remains to be established precisely where in the reaction cycle it occurs. To address this question we examine the conformational equilibrium of CPR in solution using small-angle neutron scattering (SANS) and transient kinetics. The power of solution scattering in studying domain organisation in proteins is well established; by using SANS rather than SAXS we are able to control the redox state of the enzyme precisely without the problems associated with reduction of the flavins by X-ray-induced photo-electrons^37^. These experiments allow us to relate the domain movement to individual steps in the catalytic cycle of the enzyme.

## Results

### Effects of redox state on CPR conformation

**Figure 2:**
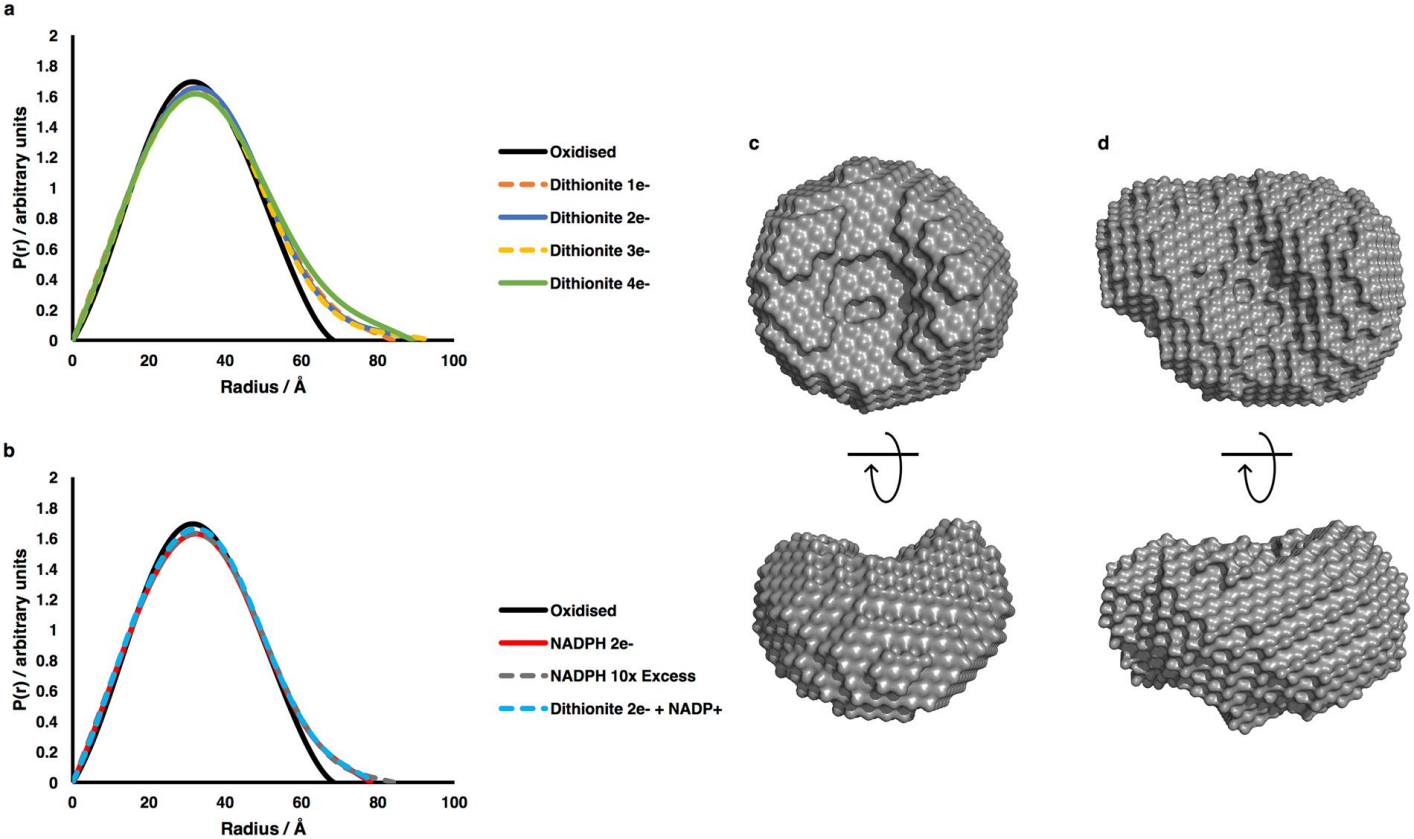
Structural information from SANS data on the different redox states of CPR. (**a**,**b**) Pairwise distance distribution functions; (**a)** samples reduced with dithionite; (**b**) samples reduced with NADPH. The longer ‘tail’ on the distribution functions for the reduced states as compared to the oxidised state demonstrates a more extended average conformation. (**c**,**d**) *ab initio* envelopes calculated using DAMMIF from the scattering curves of (**c**) oxidised CPR and (**d**) CPR reduced to the 2e^−^ level with dithionite; in (**c** and **d**) the lower envelopes have been rotated 90° about the horizontal axis.

Figure 2a,b shows the intraparticle distance distribution function, P(*r*), derived from the neutron scattering (SANS) curves, for each state of the enzyme studied, while Table 1 summarises the radii of gyration, R_g_, and the maximum dimensions, D_max_, for each state. (The scattering curves and the derived Guinier plots are shown in the Supplementary Material; Supplementary Figure 2).

**Table 1:** SANS data for different redox states of CPR; Derived hydrodynamic parameters and analysis in terms of two-state models.

Sample	Hydrodynamic parameters		Two-state Models^a^					
	R_g_, Å	D_max_^b^, Å	Crystal structure + Huang *et al*. model			Crystal structure + ΔTGEE mutant model		
			*f*_compact_^c^	*f*_extended_^d^	χ^2^	*f*_compact_^c^	*f*_extended_^d^	χ^2^
Oxidised	24.7 ± 0.1	71	0.90	0.10	1.64	0.90	0.10	1.75
Dithionite 1e^−^-reduced	27.6 ± 0.6	84	0.67	0.33	3.12	0.48	0.52	1.99
Dithionite 2e^−^-reduced	28.6 ± 0.4	90	0.61	0.39	3.27	0.56	0.44	1.81
Dithionite 3e^−^-reduced	27.6 ± 0.5	94	0.69	0.31	2.25	0.59	0.41	1.87
Dithionite 4e^−^-reduced	27.6 ± 0.5	89	0.66	0.34	2.64	0.54	0.46	2.03
Dithionite 2e^−^-reduced + NADP^+^	26.8 ± 0.4	79	0.70	0.30	2.55	0.59	0.41	2.21
NADPH 1 equiv. (2e^−^-reduced)	27.1 ± 0.4	78	0.69	0.31	2.74	0.55	0.45	1.99
NADPH excess	26.9 ± 0.3	84	0.66	0.34	3.07	0.47	0.53	1.60

^a^The models used to analyse the scattering data in terms of a two-state equilibrium are described in the text. In both cases the compact state is described by the crystal structure of oxidised CPR; the extended structure is described *either* by the model of Huang *et al*.^37^ *or* by the structure of the ΔTGEE mutant^42^. The goodness-of-fit to the scattering curve is given by the χ^2^ statistic.

^b^All D_max_ values, determined from P(r) fits using GNOM in Primus, as part of the ATSAS suite were rated as “good” (0.8) fits or better. All errors < 2 Å.

^c^Fraction of the compact conformation.

^d^Fraction of the extended conformation.

To separate the effects of coenzyme binding and of flavin reduction, we studied the effects of reduction of CPR by dithionite as well as by NADPH. It is clear from Fig. 2 and Table 1 that reduction of CPR with dithionite leads to an elongation of the average shape of the enzyme, with increases in the observed R_g_ and D_max_ and the appearance of a clear ‘tail’ on the distance distribution function. This is true for all the levels of reduction studied, with significant differences between a number of the reduced species. The largest effect in terms of R_g_ is seen for reduction by dithionite to the 2-electron level, corresponding to the CPR^2e−^ intermediate in the catalytic cycle (see Fig. 5 below). By contrast, reduction to the 2-electron level with NADPH, corresponding to the CPR^2*e*–^ NADP^+^ intermediate, has a smaller effect on the shape of the enzyme; essentially the same results are obtained for NADPH-reduced CPR and for dithionite-reduced CPR with bound NADP^+^, showing that coenzyme binding makes the reduced enzyme more compact. These changes in shape on reduction and on coenzyme binding are illustrated in Fig. 2c,d by *ab initio* low-resolution models calculated from the scattering curves.

**Figure 3:**
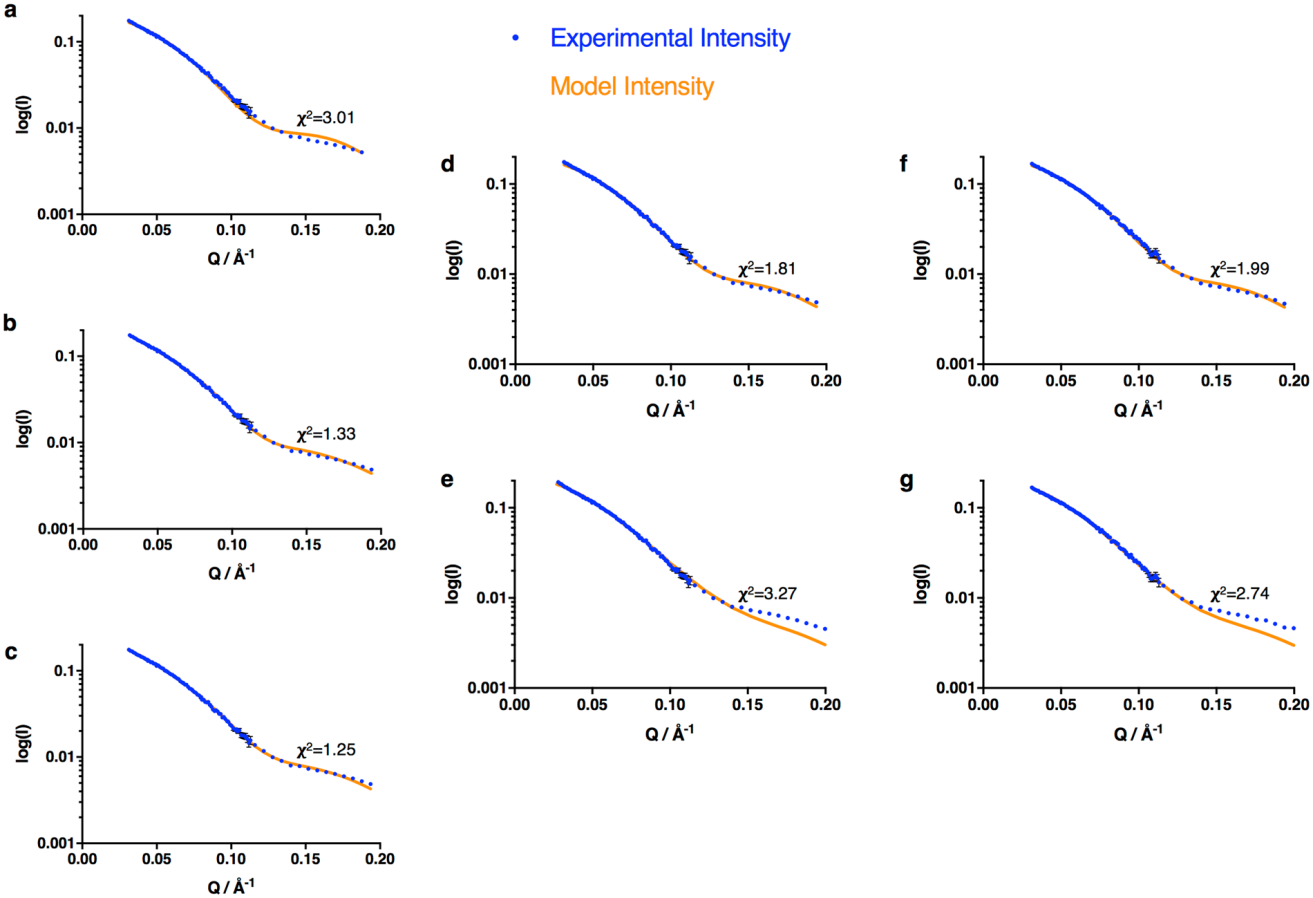
Analysis of SANS data in terms of multiple states. (**a**–**c**) Fits to the scattering curve for CPR reduced to the 2e^−^ level with dithionite using one, two or three states (from a 10,000 conformation pool) respectively. (**d**,**e**) Fits to the scattering curve for CPR reduced to the 2e^−^ level with dithionite using a two-state model; the extended state was represented by, (**d**) the model of Hamdane *et al*.^42^ or, (**e**) the model of Huang *et al*.^37^; in both cases the crystal structure was used as a model for the compact state. (**f**,**g**) Fits to the scattering curve for CPR reduced to the 2e^−^ level with NADPH using a two-state model; the extended state was represented by (**f**) the model of Hamdane *et al*.^42^ or (**g**) the model of Huang *et al*.^37^; in both cases the crystal structure was used as a model for the compact state. In all cases the goodness-of-fit is indicated by the χ^2^ value.

The results presented in Fig. 2 and Table 1 are averages over the conformational ensemble of the enzyme and the models in Fig. 2c,d do not necessarily represent single conformations. In view of the evidence cited above that CPR exists in solution as a mixture of conformational states, we have investigated whether the SANS data are better explained by such mixtures. Because of the limited information content of scattering curves and the danger of ‘over-parameterisation’, we adopt a parsimonious approach, exploring the possibility that just two conformations could fit the data adequately. Using a modification of the program MultiFoXS^39, 40^, a pool of 10,000 conformations of CPR was generated and MultiFoXS was used to select from this the single conformation or the mixture of 2, 3, … conformations which best fit the SANS data. Figure 3a–c shows that a mixture of 2 conformations was able to fit the scattering curve for 2e^–^-reduced CPR much better than a single conformation, while little improvement in fit was obtained by using a mixture of 3 conformations.

We next attempted to fit the data by using specific structural models. The compact state was represented by the crystal structure of soluble (N-terminally truncated) oxidised human CPR^19^; the R_g_ value calculated from this structure using CRYSON^41^ is 24.88 Å. To represent the more extended state we used either the model we described earlier^37^ (calculated R_g_ 30.36 Å), which was based on NMR and SAXS data on wild-type CPR, or the crystal structure of the ΔTGEE mutant of CPR, which has a deletion in the flexible hinge^42^ (PDB 3ES9); in molecule A of the crystal structure of the mutant the FMN domain has rotated away from the linker and FAD domains so as to expose the FMN to the solvent (calculated R_g_ 26.91 Å). Analysis using either of these models for the extended state, while allowing the ratio of compact to extended states to vary, gave reasonable fits to the scattering curves for all the samples studied (Fig. 3d–g; Table 1).

The SANS data for the oxidised enzyme indicated that this is almost wholly in a conformation corresponding to the crystal structure of the truncated enzyme^19^; this single conformation fits the scattering curve with a χ^2^ value of 1.87, and the experimental estimate of R_g_ is close to that calculated from the crystal structure: 24.7 (±0.1) *vs*. 24.88 Å. Introduction of a second conformation produces only a marginal increase in the goodness-of-fit; using the specific structural models for the extended state described above leads to a fit with 90% of the compact state and 10% of the extended state, giving χ^2^ values of 1.75 and 1.64 (Table 1). Even using a pool of 10,000 conformations, the MultiFoXS fit shows a population of 94% of a compact conformation closely similar to the crystal structure.

Analysis of the SANS data in terms of these two-state models shows that the increase in R_g_ and D_max_ on reduction can be accounted for by an increase in the population of the extended state (Table 1); this is observed in all the different reduced states studied, but there are some significant differences between them. In the dithionite 2e^−^-reduced state ~40% of the enzyme is calculated to be in the extended conformation. This is true whichever model is used for the extended state; the model of Hamdane *et al*.^42^ gives a somewhat better fit to the scattering curve at high *q* values, but the resolution of the SANS data does not allow us to distinguish definitively between these two models. Reduction to the same level with NADPH rather than dithionite has a significantly smaller effect when describing the extended state by the model of Huang *et al*.^37^ but not when using that of Hamdane *et al*.^42^. This difference was also noted above in terms of the average shape of the enzyme.

### Effects of ionic strength on domain motion and catalysis

**Figure 4:**
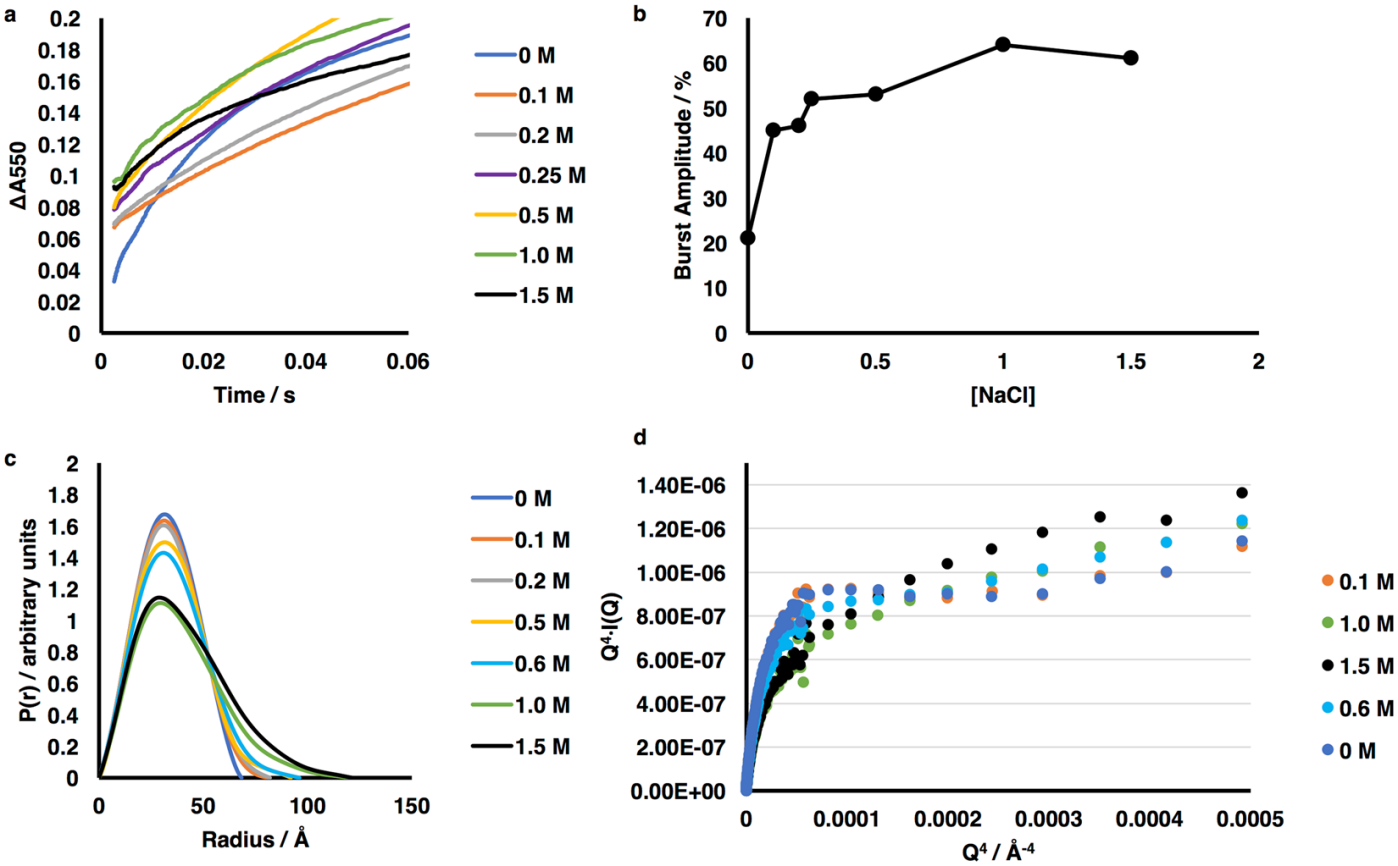
Effects of salt concentration on the kinetics of ET by CPR and on its conformation. (**a**) Stopped-flow traces showing the reduction of cytochrome c on rapid mixing of CPR pre-reduced with NADPH and cytochrome *c*, at various concentrations of added salt; the percentage of a single turnover which occurred within the 2ms deadtime of the instrument is plotted as a function of salt in (**b**). (**c**) Pairwise distance distribution functions derived from the SANS data at different salt concentrations, showing that increasing the salt concentration leads to a more extended conformation, in qualitative agreement with SAXS studies^37, 45^. (**d**) Porod-Debye plots, showing increased flexibility of CPR at ≥0.6 M NaCl.

Increasing ionic strength affects the rates of CPR-catalyzed reduction of P450s or of cyt *c* ^37, 43–45^, leading to an increase in catalytic rate, *k*_*cat*_, and in the Michaelis-Menten constant, K_M_, for cyt *c*. Kinetic traces from rapid-mixing experiments at different salt concentrations are shown in Fig. 4a. Haque *et al*.^46^ have shown that rapid mixing of CPR (pre-reduced by excess NADPH) with cyt *c* leads to a burst of cyt *c* reduction by those CPR molecules which are in a reactive (‘open’ or ‘extended’) state, followed by a slower reduction of cyt *c* by those CPR molecules that exist in a cyt *c* unreactive (‘closed’ or ‘compact’) conformation and which need to change to the ‘open’ conformation in order in interact with cyt *c*. At low salt 21% reduction of cyt *c* takes place within the 2ms dead-time (Fig. 4b), and this is in reasonable agreement with the analysis of the SANS results obtained in the presence of excess NADPH, using the model of Huang *et al*.^37^ for the extended conformation (Table 1).

As the salt concentration is increased, there is a clear increase in the fraction of the reduction taking place in the burst phase; on addition of 0.1 M NaCl, there is an increase in the fraction of reduction in the dead time to ~45%. Further increase in salt concentration leads to modest further increases in the fraction of reduction in the dead time, to ~60% at 1.5 M. Thus the kinetic results with CPR reduced by excess NADPH suggest that the fraction of the extended conformation is increased by the addition of salt. Some 50ms after mixing, the rate of cyt *c* reduction decreases to the steady-state rate, which is also clearly affected by added salt, first increasing as the salt concentration is increased, reaching a maximum at ~0.5 M NaCl, and then decreasing as the salt concentration is further increased (Supplementary Figure 3).

SANS data (Fig. 4c, Supplementary Figure 4) obtained under conditions of defined redox state show that R_g_ and D_max_ increased with increasing salt concentration (Table 2), with a gradual increase in R_g_ and D_max_ up to 0.5 M added NaCl and a considerably more marked increase thereafter. Porod-Debye plots^47, 48^ of the scattering data (Fig. 4d) indicate that there is a marked increase in the flexibility of CPR at salt concentrations of 0.6 M and above, raising the possibility of partial unfolding of the enzyme at these high salt concentrations. Analysis of the data in terms of a two-state equilibrium between compact and extended conformations was therefore restricted to data between zero and 0.5 M added salt. The fitting parameters are given in Table 2; the proportion of the extended conformation increases with salt concentration within this range, and again both models for the extended sate give essentially the same results. Thus, the SANS data show that the proportion of the extended conformation increases with increasing ionic strength, and comparison with the stopped-flow kinetic data suggests that this conformation has higher activity for cyt *c* reduction than does the compact conformation.

**Table 2:** SANS data for CPR at different salt concentrations; Derived hydrodynamic parameters and analysis in terms of two-state models.

Added NaCl, M	Hydrodynamic parameters		Two-state Models^a^					
	R_g_, Å	D_max_^b^, Å	Crystal structure + Huang *et al*. model			Crystal structure + ΔTGEE mutant model		
			*f*_compact_^c^	*f*_extended_^d^	χ^2^	*f*_compact_^c^	*f*_extended_^d^	χ^2^
0	24.7 ± 0.1	71	0.90	0.10	1.64	0.90	0.10	1.75
0.1	25.8 ± 0.1	80	0.86	0.14	2.27	0.84	0.16	2.37
0.2	25.9 ± 0.2	81	0.85	0.15	2.24	0.85	0.15	2.38
0.5	26.6 ± 0.1	91	0.72	0.28	2.12	0.72	0.28	2.52
0.6	27.4 ± 0.2	96						
1.0	30.3 ± 0.3	119						
1.5	31.6 ± 0.4	121						

^a^The models used to analyse the scattering data in terms of a two-state equilibrium are described in the text. In both cases the compact state is described by the crystal structure of oxidised CPR; the extended structure is described *either* by the model of Huang *et al*.^37^ *or* by the structure of the ΔTGEE mutant^42^. The goodness-of-fit to the scattering curve is given by the χ^2^ statistic. The two-state models were not used to analyse the data for >0.5 M added salt; see text.

^b^All D_max_ values, determined from P(r) fits using GNOM in Primus, as part of the ATSAS suite, were rated as “good” (0.8) fits or better. All errors < 2 Å.

^b^Fraction of the compact conformation.

^c^Fraction of the extended conformation.

## Discussion

The solution scattering data presented here provide unambiguous evidence that the movement of domains of CPR relative to one another is affected by redox state of the flavins and by coenzyme binding. In turn, perturbation of the conformational equilibrium affects the ET to cytochrome *c*. Together, these experiments demonstrate orchestrated domain movements in the catalytic cycle of CPR.

### The nature of the conformational equilibrium

The most parsimonious description of CPR in solution is a *two*-*state* equilibrium between compact & extended conformations, the two states being similar for all the states of CPR studied, and this provides a good framework within which to discuss the importance of domain movement in CPR.

The crystal structures of wild-type rat, yeast and human CPR^17, 19, 49^ all show a compact conformation, with the isoalloxazine rings of FAD and FMN in close proximity as would be required for inter-flavin ET. The crystal structure of the human enzyme accounts for the SANS data on oxidised CPR in solution reasonably well, although the data are fit slightly better if ~10% of an extended model is included. Consistent with the present SANS data, Vincent *et al*.^50^ concluded from NMR experiments that oxidised CPR is 95% in the compact state.

Two structural models of the extended state^37, 42^ can account for the SANS data within the framework of a two-state model. Both these models for the extended state are consistent with the observation that two mutants in which the inter-domain salt bridges K75-E354 and R78-D352 (residue numbering throughout corresponds to that for the human enzyme used in our earlier work)^37^ seen in the compact conformation are abolished show an increased population of the extended conformation^37, 51^, since in both models these pairs of residues are distant from one another. Scrutton’s laboratory has used FRET measurements between dyes attached to the naturally occurring cysteine residues in the FMN and FAD domains to study domain movement^38^. The results were discussed in terms of a model in which the compact (‘closed’) form of the enzyme corresponds to the crystal structure and the extended (‘open’) form to the structure of the ΔTGEE mutant^42^; the positions of the cysteines are such that the transition to this extended form would lead to an *increase* in the FRET signal^38^. Hedison *et al*. propose that the oxidised form of the enzyme is predominantly in the extended conformation and that coenzyme binding and ET lead to successively greater proportions of the compact form^38^. This is not consistent with the SANS results presented here, which show clearly that the oxidised enzyme is almost wholly in the compact state; the same conclusion has been reached by NMR^50^ and mass spectrometry^51^. However, these FRET experiments are entirely consistent with the structural experiments if the model for the extended state is taken to be that of Huang *et al*.^37^, since in this model the FRET signal between the dye-labelled cysteine residues would be expected to *decrease* as the proportion of the extended form increased. Recently, Kovrigina *et al*.^52^ have studied the domain movement by measuring FRET between dyes attached to two specifically engineered cysteines; consistent with our present results, they concluded that oxidised CPR is in a *compact* conformation. The NMR and FRET results thus lead to a slight preference for the model of Huang *et al*.^37^ for the extended state. However, it should be emphasised that the model based on the ΔTGEE mutant fits the SANS data slightly better, and this mutant forms a stable complex with heme oxygenase^53^.

### Relation to the catalytic cycle

**Figure 5:**
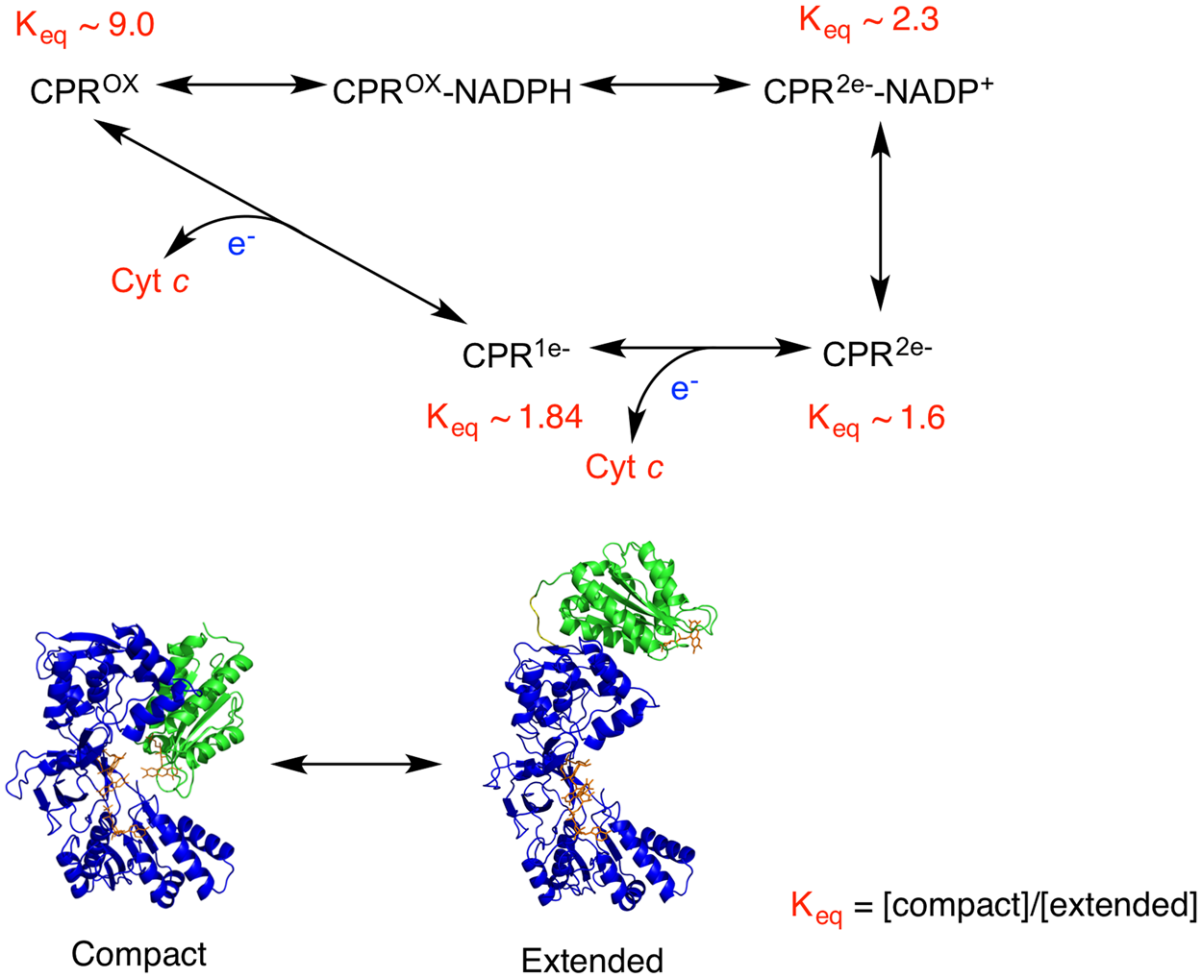
Catalytic cycle of CPR, showing the position of the conformational equilibrium for each intermediate. The reaction shown is cytochrome *c* reduction *in vitro*, as discussed in the text. For illustrative purposes, the compact state is represented by the crystal structure of the oxidised enzyme^19^, and the extended conformation by the model of Huang *et al*.^37^.

The catalytic cycle of CPR for an *in vitro* reaction starting with the fully oxidised enzyme is shown in Fig. 5; NADPH binds to the FAD domain where it transfers a hydride ion to the N5 of FAD, followed by ET from FAD to FMN to yield a quasi-equilibrium distribution of 2e^−^-reduced species. Analysis of equilibrium redox titration data^54^ led to an approximate estimate of [FAD-FMNH_2_]/[FAD•-FMN•]~11, with only a small amount of FADH_2_-FMN. Intermolecular ET to cyt *c* takes place from FMNH_2_^18^, and then probably from FMN•^55^. This represents a 0-2-1-0 cycle of redox states (in terms of numbers of electrons). It has been suggested that *in vivo* the ‘resting state’ of CPR is a 1e^−^-reduced (FAD-FMN•) state, reduction by NADPH leading to a 3e^−^-reduced state and ET to cytochrome P450 taking place only from FMNH_2_ – that is a 1-3-2-1 redox cycle^18^. An unambiguous choice between these two cycles cannot yet be made.

SANS allows us to relate our results on domain movements to the catalytic cycle secure in the knowledge that the redox state of CPR is well defined in our experiments. The oxidised enzyme is essentially completely in the compact state (K_eq_ ~9; Fig. 5). In the CPR 2e^−^ - NADP^+^ species, the product of the initial hydride transfer, the proportion of the extended state is ~30%. The retention of a significant population of the compact state in this species is consistent with the obvious requirement for a conformation with the FAD and FMN in close proximity for interflavin electron transfer. Indeed, relaxation kinetics shows that the rate of interflavin electron transfer increases on binding NADP^+^^27, 28^. On the subsequent dissociation of NADP^+^, the population of the extended state increases to ~40%, facilitating electron transfer to cytochrome P450 (or cyt *c*).

### Structural triggers for domain movement

The interaction between the FMN and FAD/linker domains in CPR is weak; no interactions between the isolated FMN and FAD/linker domains are detected kinetically, spectroscopically, or by ITC^56, 57^ and the redox potentials of the separated domains are essentially the same as those of the intact enzyme^58^. Thus only small changes in interactions across the interface would be required to perturb the conformational equilibrium.

In CPR FMN N5 is positioned so that it might form a hydrogen bond to the peptide NH of G144 when the flavin is oxidized and to the carbonyl of this residue when the flavin is protonated in the reduced states. A reorientation of this peptide bond would thus be required on formation of the neutral semiquinone and protonation at N5. There is good evidence for this in structures of flavodoxins^59–61^ and in the recent comparison of the structures of oxidised and reduced rat CPR with 2′-AMP bound^62^. This ‘peptide flip’ is accompanied by changes in the neighbouring residues, notably Y143 and E145, the latter being in the inter-domain interface. Although the enzyme was reduced in preformed crystals^62^, possibly inhibiting domain movement, there is a clear change in the relative position of the domains on reduction in one of the two molecules in the asymmetric unit. As we noted^37^, the electron density map of the X-ray structure of human CPR^19^ supports the same role for G144 in the human enzyme; density can clearly be seen that shows this peptide bond in two positions.

Coenzyme binding appears to be a two-step process^18, 29, 63, 64^: first the 2′5′-ADP part binds, then – associated with a displacement of W679 which is stacked against the isoalloxazine ring of FAD – the nicotinamide moves into place next to the FAD. In the structure of the W679/S680 deletion mutant^65^ there is a disordered FMN domain in one molecule of the asymmetric unit, suggesting involvement of these residues in determining the relative orientation of the domains. Indeed, in the structure of the human enzyme there are water-mediated hydrogen-bonds between the C_β_-OH of S680 and N178 and D212 in the FMN domain^19^. There is a hydrogen bond between the backbone of W679 and that of D634, in a flexible loop comprising residues G633-N637; R636 in this loop hydrogen-bonds to T180 in the FMN domain. This loop is close to the adenine ring of the bound coenzyme, and in the structures of the disulphide cross-linked mutant^29^ it moves on NADP^+^ binding; mutagenesis studies support a role for this loop in coenzyme binding and flavin reduction^64^. It is thus likely that a concerted movement of W679/S680 and the G633-N637 loop on coenzyme binding will affect the domain interface and alter the equilibrium between the compact and extended states.

## Conclusion

It is clear that in CPR protein dynamics, and specifically domain motions, are involved in ensuring productive electronic coupling between the flavin cofactors and between them and the electron acceptor protein. The conformational search required to reach these productive configurations can limit the rate of ET^28^. We have now shown that in the case of CPR this search can be adequately described by a two-state equilibrium. We have for the first time been able to link this conformational equilibrium to the reaction cycle of the enzyme, describing its position in each of the key intermediate states.

## Materials

NADPH, NADP^ +^ , dithionite, potassium ferricyanide and horse heart cytochrome *c* were purchased from Sigma-Aldrich. All other chemicals were of analytical grade.

### Protein Expression and Purification

The gene for human fibroblast CPR lacking the N-terminal membrane-anchoring region (a kind gift from Professor C.R. Wolf, University of Dundee) was expressed in *Escherichia coli* BL21 STAR cells using the pCS22 (cold-shock) plasmid construct^66^. Cells were grown to the mid-log phase in TB medium at 37 °C prior to induction by reducing the growth temperature to 15 °C to exploit the cold-shock promoter. CPR was purified as described previously^56, 66^, with modifications. The purification involved use of a 2′5′-ADP affinity column; the pure protein was eluted using a 20% glycerol solution rather than 2′-AMP in order to avoid undesired persistent binding of the 2′-AMP. A final stage of purification included the use of size exclusion liquid chromatography in order to isolate the purely monomeric form of the protein, essential in small angle scattering experiments. The protein concentration was calculated using a molar extinction coefficient of ε_450_ = 22,000 M^−1^cm^−1^.

### Cytochrome c Reduction Assays

Steady-state cytochrome *c* reduction assays following absorbance change at 550 nm were carried out in 100 mM BES [N,N-bis(2-hydroxyethyl)-2-amino-ethane sulfonic acid], pH 7.0, with 50 μM cytochrome *c* and 50 μM NADPH at 25 °C. Burst-phase kinetics of the reduction of cytochrome *c* by fully reduced CPR was studied by stopped-flow under anaerobic conditions at 10 °C. The stopped-flow apparatus (Applied Photophysics, UK) was placed inside a glovebox (Belle Technology, UK) in an atmosphere with an oxygen content of 5 ppm or less. All transient kinetics studies were carried out in 100 mM BES pH 7.0 buffer. A solution containing 10 μM CPR and 200 μM NADPH was incubated for 5 minutes in anaerobic conditions before starting any measurements. The reduced protein solution was rapidly mixed with an equal volume of 100 μM cytochrome c in the 2 μL flow cell and the change in absorbance at 550 nm after the 2 ms dead-time of the instrument was recorded. 2000 data points were measured over a time period of 1 s in order to ensure that the full burst phase, as well as the transition to the slow steady-state phase was observed. In order to provide an initial reading for A_550_ in the absence of reduction, the cytochrome *c* solution was also mixed with the buffer solution only.

### Redox Titrations

The spectrophotometric recording of the reduction of CPR with sodium dithionite or NADPH was carried out at 25 °C using a Jasco V-730 spectrophotometer in a glovebox (Belle Technology, UK) in a nitrogen atmosphere with < 5ppm oxygen. All samples were in 100 mM BES, pH 7.0, buffer. All solutions were purged in a nitrogen atmosphere before introduction to the glovebox and then incubated for 4–6 hours on ice before continuing. The reducing agent titrants were prepared inside the glovebox using degassed buffer and their concentration determined using ε_315_ = 8040 M^−1^ cm^−1^ and ε_340_ = 6220 M^−1^ cm^−1^ for sodium dithionite and NADPH respectively. The redox titrations were carried out as previously described^67^. A small aliquot of the reducing agent solution was added to the protein solution and the spectrum was recorded after a period of equilibration (Supplementary Figure 1). The titrants were added stoichiometrically, recording the exact molar ratios of the reagents before ending the titrations once the 4-electron-reduced state or other relevant end-point was reached. Once the protein solution was determined to be at the desired redox state a 200 μL aliquot was taken and sealed inside a necked quartz SANS cuvette with a rubber O-ring cap and air-tight film. Samples were checked spectroscopically after the SANS measurements to confirm that the redox state remained unaltered.

### Solution Scattering Data Collection and Analysis

SANS measurements were carried out on D22, the high-flux neutron diffractometer at the Institut Laue-Langevin, Grenoble, France. The data collection parameters are given in Supplementary Table 1. Each CPR sample of 2–4 mg/mL in 100 mM BES, pH 7.0, was measured in a 1 mm path length quartz cuvette at 10 °C for a total of 1 hr in order to gather data with a suitably high statistical precision. Alternatively, the instrument was set up in on-line FPLC mode where the sample was loaded on to a size exclusion column (GE Healthcare Life Sciences, Superdex 200 Increase 10/300 GL) and SANS measurements were taken after the void volume^68^. In this case the flow rate on the FPLC (Knauer LC, Germany) was set to 0.3 mL/min and SANS measurements were taken throughout the whole protein elution time with a frame duration of 60 s in a custom made quartz flow-cell (Hellma Analytics, Germany) with a 1 mm path length.

Data were recorded at two collimation lengths (5.6 m and 2.8 m) and respective sample-to-detector distances (5.6 m and 1.4 m) in order to provide a full *q* range from the Guinier region of the monomer to the solvent. The 2-dimensional ^3^He detector was positioned at different distances from the sample with an off-centered direct beam in order to provide a q-range of 0.01–0.6 Å^−1^, where q = 4π sin θ/λ and 2θ is the scattering angle at a wavelength of 6 Å ± 10%. The raw scattering data were reduced using the instrument specific software GRASP^69^, merged to produce the full scattering curves and buffer subtracted and normalised for concentration as appropriate using NIST SANS reduction macros in IGOR pro^70^. Where samples were found to show small amounts of unavoidable aggregation, the scattering curves were fit to the Beaucage model^71^ to ensure that their influence on the Guinier region was negligible.

Initial data processing and analysis were carried out using programs from the ATSAS suite^72^. Determination of hydrodynamic parameters was performed using PRIMUS^73^, where R_g_ was determined using the Guinier approximation, and D_max_ and P(*r*) were calculated using GNOM^74^. Model-independent *ab initio* molecular envelopes were generated using DAMMIF^75^. Fifteen independent DAMMIF runs were averaged using DAMAVER^76^ to obtain a typical molecular shape and filtered using DAMFILT to produce a refined model revealing only the most common structural features.

Rigid body modelling was carried out using software from the IMP (Integrative Modelling Platform) suite^77^ and the ATSAS suite. Either a pool of specific structural models, as discussed in the text, or a pool of 10,000 conformational samples created using the RRT (rapidly exploring random tree) sampling tool provided with a PDB structure and a selection of flexible residues, were used. For the specific structural models, the compact state was represented by the crystal structure of soluble (N-terminally truncated) oxidised human CPR^19^; the R_g_ value calculated from this structure using CRYSON^41^ is 24.88 Å. To represent the more extended state we used either the model we described earlier^37^ (calculated R_g_ 30.36 Å), which was based on NMR and SAXS data on wild-type CPR, or the crystal structure of the ΔTGEE mutant of CPR^42^ (PDB 3ES9). In the crystals of this mutant, which has a deletion in the flexible hinge^42^, there are three molecules in the asymmetric unit, in each of which the FMN domain has moved relative to the linker and FAD domains. In molecule A the position of the FMN domain is well-defined and can be seen to have rotated away from the linker and FAD domains in such a way as to expose the FMN to the solvent, with a distance between the two isoalloxazine rings of ~29 Å; we have used molecule A as a model for the extended state (calculated R_g_ 26.91 Å).Theoretical scattering curves were calculated for each of the sampled conformations using CRYSON^41^. A best fit model to the experimental data was determined using MultiFoXS^40^ in partial mode where precomputed scattering intensities were used. For most fits the maximum q-value was set at 0.2 Å^−1^ due to the levels of experimental noise present in the high-q data. The best-scoring models comprising one or more states from the pool provided were identified by using the χ^2^ value describing the goodness-of-fit to the experimental data.

## Electronic supplementary material

Supplementary Information
