# Supplementary material for: Orchestrated Domain Movement in Catalysis by Cytochrome P450 Reductase
Source: Sci Rep. 2017 Aug 29;7:9741. doi: 10.1038/s41598-017-09840-8 (PMC5575293; doi:10.1038/s41598-017-09840-8)
Supplement: Supplementary file 1 — Supplementary Information [file 41598_2017_9840_MOESM1_ESM.pdf]

Orchestrated Domain Movement in Catalysis by Cytochrome P450 Reductase

Samuel L. Freeman, Anne Martel, Emma L. Raven and Gordon C.K. Roberts

SUPPLEMENTARY MATERIAL

Supplementary Table 1 – SANS Parameters

**Data Collection**	
Instrument	ILL D22
Wavelength (Å)	6 (± 10%)
Q range ($Å^{-1}$)	0.01 – 0.6
Total exposure time (min)	60
Temperature (K)	283
Concentration range (mg ml^{-1})	1 – 5
**Structural parameters** [a]	
I(0) (cm^{-1}) (from P(*r*))	0.151
I(0) (cm^{-1}) (from Guinier)	0.15 ± 0.00044
R_g (Å) (from P(*r*))	24.69 ± 0.13
R_g (Å) (from Guinier)	24.71 ± 0.12
D_{max} (Å)	70.33
Porod volume estimate ($Å^3$)	62221
**Molecular mass**	
Molecular mass M_r, from I(0)	68,800
Calculated monomeric M_r	69,585
**Software employed**	
Primary data reduction	GRASP(1)
Data processing	NIST SANS reduction macros(2)<br>GNOM/PRIMUS(3, 4)
Ab initio analysis, validation & averaging	DAMMIF/DAMAVER(5, 6)
Rigid-body modelling	MultiFoXS / rrt_sample(7, 8)
Computation of model intensities	CRYSON(9)

[a] Reported for the oxidised enzyme at 2 mg ml^{-1}

Supplementary Figures

Supplementary Figure 1

Superimposed optical spectra of CPR on incremental anaerobic titration with dithionite. Coloured lines indicate samples used for SANS measurements of defined redox states.

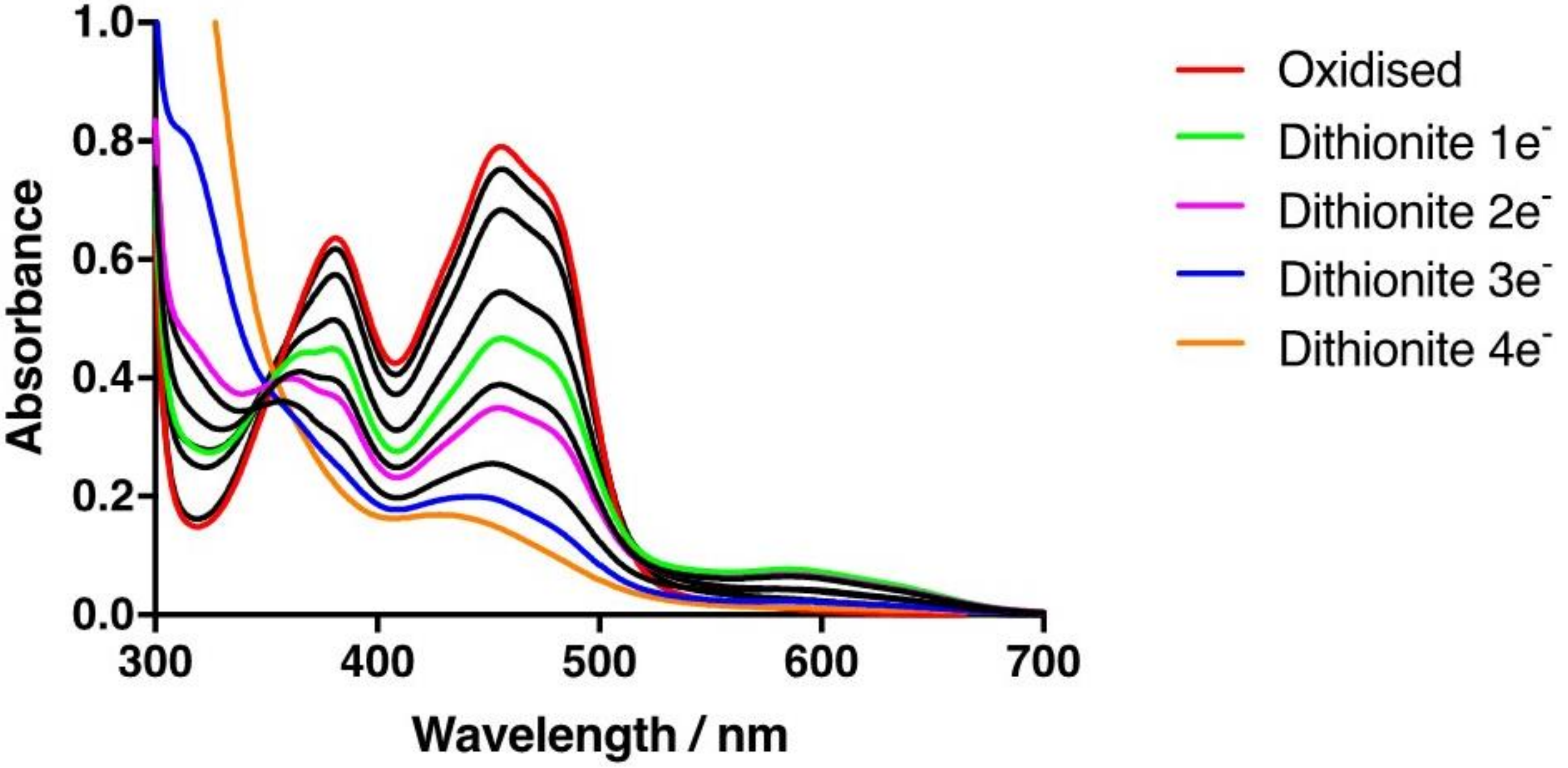

Supplementary Figure 2.

SANS scattering curves (a) and Guinier plots (b) for CPR in different defined redox states. In both cases the different curves have been displaced vertically for clarity.

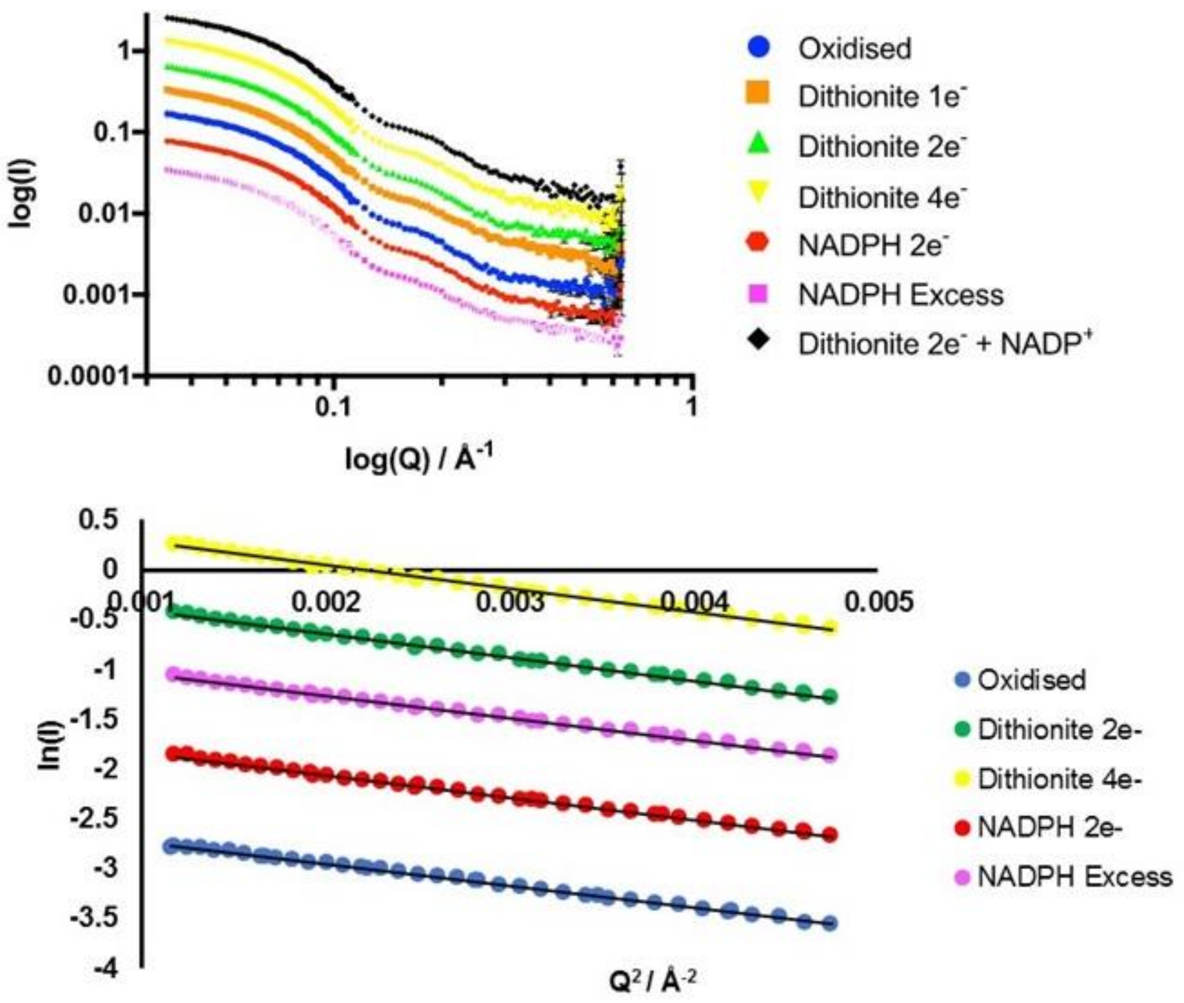

Supplementary Figure 3.

Steady-state rate of reduction of cytochrome c by NADPH-reduced CPR as a function of added salt concentration. The increase with salt concentration up to ~0.5M added salt, followed by a decrease at higher concentration is consistent with earlier reports(10, 11).

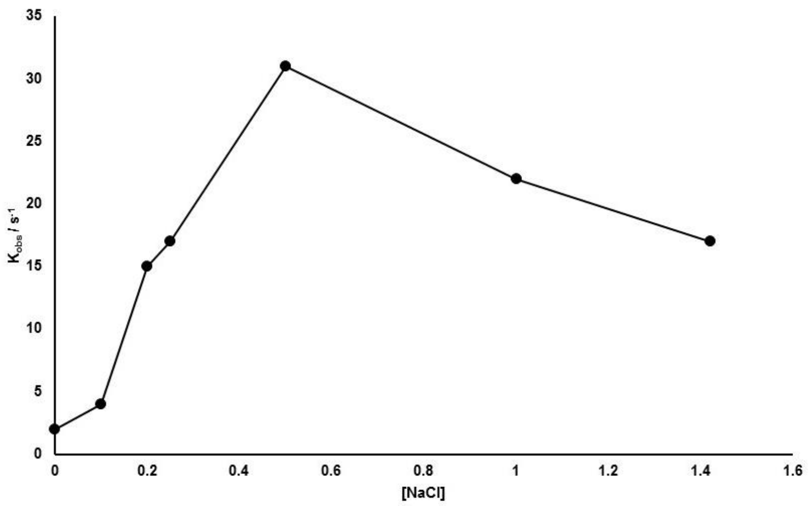

Supplementary Figure 4.

SANS scattering curves (a) and Guinier plots (b) for CPR at different concentrations of added salt. In both cases the different curves have been displaced vertically for clarity.

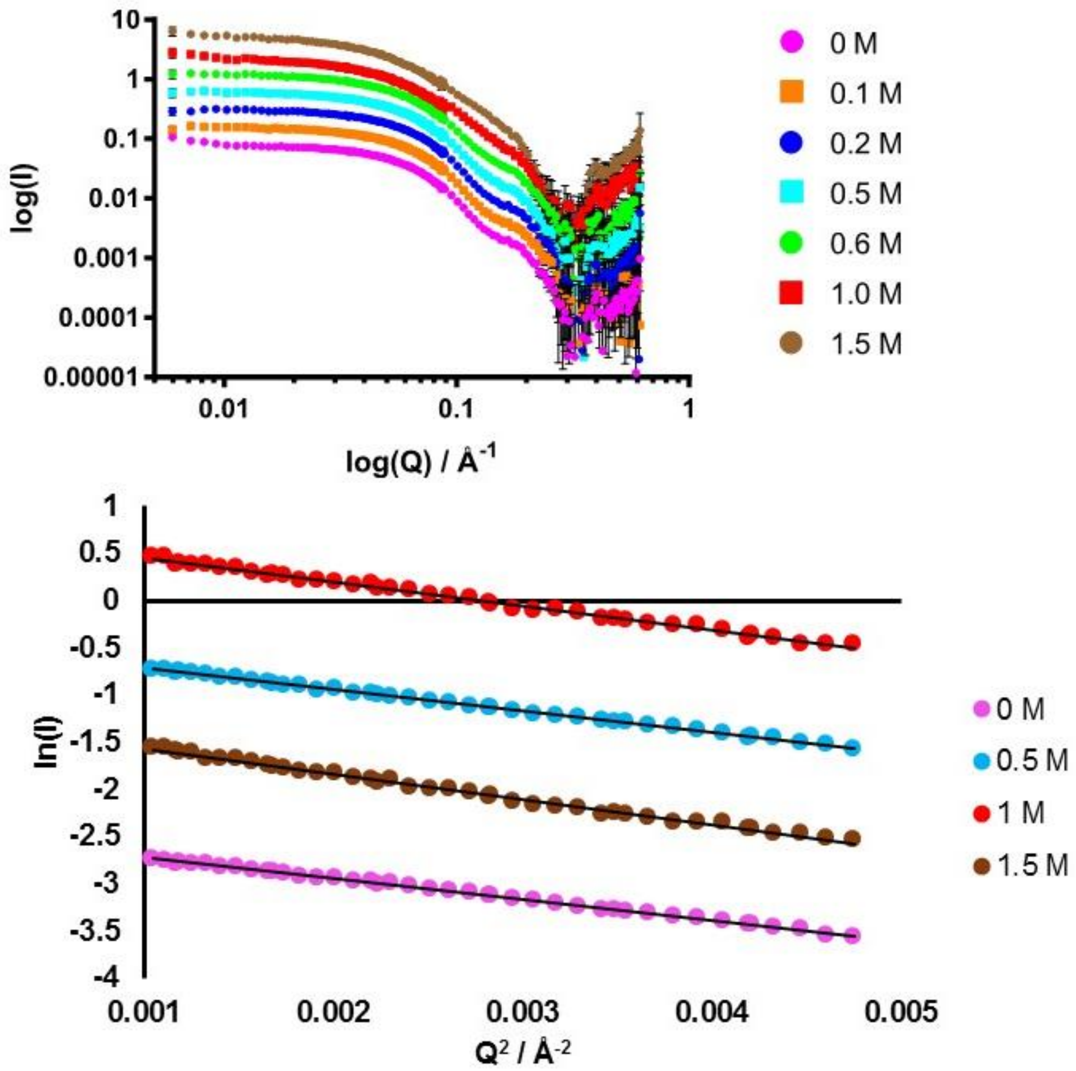

References

1. ILL (2016) ILL-D22 Documentation 2016
2. Kline SR (2006) Reduction and analysis of SANS and USANS data using IGOR Pro. *Journal of Applied Crystallography* 39(6):895-900.
3. Konarev P, Volkov, V., Sokolova, A., Koch, M., and Svergun, D. (2003) PRIMUS : a windows PC-based system for small-angle scattering data analysis. *J. Appl. Crystallogr.* 36(5):1277-1282.
4. Svergun D (1992) Determination of the regularization parameter in indirect-transform methods using perceptual criteria. *Journal of Applied Crystallography* 25(4):495-503.
5. Franke D & Svergun DI (2009) DAMMIF, a program for rapid ab-initio shape determination in small-angle scattering. *J Appl Crystallogr* 42(Pt 2):342-346.
6. Volkov VV, and Svergun, D. I. (2003) Uniqueness of ab initio shape determination in small-angle scattering. . *J. Appl. Crystallogr.* 36(3-1):860-864.
7. Russel D*, et al.* (2012) Putting the pieces together: integrative modeling platform software for structure determination of macromolecular assemblies. *PLoS Biol* 10(1):e1001244.
8. Schneidman-Duhovny D, Hammel M, Tainer JA, & Sali A (2016) FoXS, FoXSDock and MultiFoXS: Single-state and multi-state structural modeling of proteins and their complexes based on SAXS profiles. *Nucleic acids research* 44(W1):W424-W429.
9. Svergun DI, Richards, S., Koch, M.H.J., Sayers, Z., Kuprin, S., and Zaccai, G. (1998) Protein hydration in solution: Experimental observation by x-ray and neutron scattering. *Proc. Natl. Acad. Sci. USA* 95(5):2267-2272.
10. Frances O*, et al.* (2015) A well-balanced preexisting equilibrium governs electron flux efficiency of a multidomain diflavin reductase. *Biophys J* 108(6):1527-1536.
11. Huang WC, Ellis J, Moody PC, Raven EL, & Roberts GC (2013) Redox-linked domain movements in the catalytic cycle of cytochrome p450 reductase. *Structure* 21(9):1581-1589.
